# Association between the sarcopenia index and the risk of stroke in elderly patients with hypertension: a cohort study

**DOI:** 10.18632/aging.204587

**Published:** 2023-03-21

**Authors:** Xintian Cai, Junli Hu, Mengru Wang, Wen Wen, Jingyu Wang, Wenbo Yang, Yujie Dang, Qin Luo, Jing Hong, Nanfang Li

**Affiliations:** 1 Hypertension Center, Xinjiang Hypertension Institute, NHC Key Laboratory of Hypertension Clinical Research, Key Laboratory of Xinjiang Uygur Autonomous Region, Xinjiang Clinical Medical Research Center for Hypertension Diseases, People’s Hospital of Xinjiang Uygur Autonomous Region, Ürümqi, Xinjiang 830000, China; 2 Renal Division, Peking University First Hospital, Beijing 100034, China

**Keywords:** sarcopenia index, hypertension, elderly patients, stroke, cohort study

## Abstract

The purpose of this study was to investigate the relationship between the sarcopenia index (SI) and stroke risk in elderly patients with hypertension. This study included 5145 stroke-free elderly hypertensive patients. We used Cox regression models to estimate hazard ratios (HRs) and 95% confidence intervals (CIs) of incident stroke. Over a median follow-up of 38 months, we identified 607 (11.80%) individuals with total stroke, of whom 507 (9.85%) had ischemic stroke and 93 (1.81%) had hemorrhagic stroke. The risk of developing stroke decreased with each quartile of SI; after adjustment for multiple confounders, the HRs for the Q4 group versus the Q1 group were 0.46 (95% CI, 0.35–0.59) for total stroke, 0.46 (95% CI, 0.35–0.61) for ischemic stroke, and 0.33 (95% CI, 0.17–0.64) for hemorrhagic stroke. Restricted cubic spline analysis also demonstrated a cumulative increase in the risk of total stroke with decreases in the SI. The addition of SI to the conventional model for total stroke improved (ΔC-statistics = 0.02), an integrated discrimination improvement of 0.03 (95% CI, 0.02–0.04), and a net reclassification improvement of 0.17 (95% CI, 0.10–0.23). Similar results were observed for ischemic stroke and hemorrhagic stroke. This study found that elevated SI was negatively associated with the risk of stroke in elderly patients with hypertension. Uncovering the causality behind the relationship requires further prospective study.

## INTRODUCTION

Stroke has become a major public health problem worldwide, and the financial burden associated with stroke treatment and post-stroke care is significant, especially for older adults [1, 2]. Stroke is a common cause of death and disability in the aging population [3]. In China, stroke was the leading cause of death, disability-adjusted life years, and years of life lost in 2017 [2]. Hypertension is the most common risk factor for patients with prevalent stroke in China [4]. In addition, traditional risk factors cannot fully explain all the risks of stroke [5–7]. Therefore, it is clinically important to understand more modifiable risk factors for stroke, especially in older patients with hypertension.

Sarcopenia is a significant geriatric condition that affects aging societies and is characterized by decreased skeletal muscle mass, low muscular strength, and/or poor physical performance [8]. According to several studies, the percentage of elderly Asian individuals who have sarcopenia ranges from 6.8% to 25.7% [9–12]. Many studies have indicated that sarcopenia and heart disease in the elderly have many pathophysiological aspects in common [13–15]. Sarcopenia is independently associated with prevalent cardiovascular diseases and their associated risk factors [16–18]. Currently, muscle mass is quantified using magnetic resonance imaging, computed tomography, dual-energy X-ray absorptiometry, and bioelectrical impedance analysis [19–21]. However, these techniques have drawbacks such as radiation exposure and a lack of cost effectiveness, as well as increased needs for skilled experts and specialized equipment [22]. It was recently suggested to use the sarcopenia index (SI), a straightforward alternative screening method based on the ratio of serum creatinine (Cr) to cystatin C (CysC) levels [23]. Recent epidemiological studies have also demonstrated that in various populations, including the elderly, SI can be effectively used to measure muscle mass, strength, and functional status [23–25]. According to Hyun et al., individuals with chronic renal disease had an increased risk of all-cause mortality and cardiovascular events when their blood Cr/CysC ratio was high [26]. A low Cr/CysC ratio has also been linked to cardiovascular disease events and death in older individuals with coronary artery disease and those with obstructive coronary artery disease [27, 28]. However, little research has been done to investigate the association between SI and new-onset strokes. To date, the relationship between SI and the risk of stroke is still unknown, especially in older patients with hypertension.

Therefore, we conducted a cohort study to investigate the association between SI and the risk of stroke in elderly patients with hypertension.

## MATERIALS AND METHODS

### Patient selection

In this retrospective cohort study, elderly hypertensive patients (age ≥ 60 years) were admitted to the People’s Hospital of Xinjiang Uygur Autonomous Region. Detailed descriptions of this study have been reported previously [29]. Those who met the following criteria were excluded: (1) patients had no data on Cr or CysC, (2) patients with kidney disease or an eGFR of less than 60 mL/min/1.73 m^2^, (3) loss to follow-up or follow-up duration <6 months, and (4) patients had a history of stroke, malignancy, liver cirrhosis, advanced heart failure, or chronic lung disease. After these exclusions, 5145 participants were included in the final analysis. Participant flow is illustrated in Supplementary Figure 1. Approval was obtained from the Ethical Committee of the People’s Hospital of Xinjiang Uygur Autonomous Region (No. KY2021031901). Owing to the retrospective data collection, it was not deemed to require informed consent. We followed the Strengthening the Reporting of Observational Studies in Epidemiology (STROBE) guideline recommendations.

### Data collection and measurements

Data were abstracted electronically from the patient’s medical records, including demographics, anthropometric measures, risk factors, diagnoses according to the International Classification of Diseases 10th Revision (ICD-10), prescribed medications, and laboratory data. Smoking status was dichotomized as current smokers vs. non-smokers. A similar classification was used for alcohol use (current drinkers and non-drinkers). All blood samples were collected in the morning, on fasting participants. Laboratory parameters included fasting plasma glucose (FPG), hemoglobin A1c (HbA1c), fasting lipid profile, liver and renal function tests, homocysteine (Hcy), and high-sensitivity C-reactive protein (hs-CRP). Serum Cr level was measured using an enzymatic method, and CysC levels were measured with the immunoturbidimetric assay. The SI was calculated as (serum Cr divided by serum CysC)*100 [23]. eGFR was estimated using the Chronic Kidney Disease Epidemiology Collaboration (CKD-EPI) equation [30]. Information on disease history was obtained using ICD-10 codes. To ensure the accuracy of diagnoses, coronary heart disease (CHD) (I24 and I25), diabetes (E10-E14), atrial fibrillation (I48), and dyslipidemia (E78) were regarded as present if a participant was treated ≥ 2 times. The burden of comorbidity was measured using the Charlson Comorbidity Index (CCI) [31]. Prescription claims in the last year prior to the concomitant medications identified at baseline. The list of concomitant medications included in this study is shown in Supplementary Table 1.

### Follow-up and outcome assessment

Each participant’s follow-up period (measured in person-years) was computed from the baseline date to the earliest of the following dates: incidence of stroke, death, loss to follow-up, or 31 December 2021 (the study’s end of follow-up), whichever came first. Outcomes of events since participants enrolled in the study were determined through checking medical records, interviews, contact with local disease and death registries, and electronic linkage with the national health insurance claim databases. Trained staff with no knowledge of baseline information used ICD-10 to code all diagnoses and deaths. The primary outcome was the first occurrence of total stroke (ICD-10: I60-I64), including morbidity and mortality. Secondary outcomes included first ischemic stroke (ICD-10: I63) and first hemorrhagic stroke [subarachnoid (ICD-10: I60) or intracerebral (ICD-10: I61)]. An independent clinical events committee reviewed and centrally adjudicated these outcome events.

### Statistical analysis

Details of the missing covariates are shown in Supplementary Table 2. We performed multiple imputation to recover missing covariates. Baseline characteristics were compared between groups categorized by SI levels. The age-adjusted incidence rates were determined by calculating age-specific incidence rates within 1-year age categories. Time to first stroke event was examined using Kaplan-Meier survival curves and compared using log-rank test. Multicollinearity was tested using the variance inflation factor (Supplementary Table 3). Testing for proportional hazards used Schoenfeld residuals (Supplementary Figure 2). Cox regression analysis was used to assess the association between SI and stroke and its subtypes, and hazard ratios (HRs) with 95% confidence intervals (CIs) were estimated. Values for trend tests were assigned using the within-quartile medians. Additionally, we used restricted cubic splines to investigate the non-linear associations between SI and the outcomes. Furthermore, subgroup analyses were done and differences were examined using tests for interaction. Sensitivity analyses assessed robustness of results. First, we specified a 1-year exposure lag to circumvent the potential bias of reverse causation. Second, we did competing-risk analysis that treats non-stroke-related deaths as a competing risk. Third, sensitivity analyses were performed that excluded all individuals with CCI ≥ 2. Fourth, participants with atrial fibrillation were excluded. Lastly, potential unmeasured confounding was examined by calculating E-values. The additional value of adding SI to the conventional model was evaluated by C-statistics, the net reclassification index (NRI), and the integrated discrimination index (IDI). More details are found in the Supplementary Materials and Methods.

All analyses were performed using R 4.1.1 software, with a two-sided significance *p*-value < 0.05.

## RESULTS

### Study population and characteristics

As illustrated in the flowchart (Supplementary Figure 1), a total of 5145 eligible participants were included in the present study. Among the included participants, the average age was 66.54 ± 4.79 years, and 2481 (48.22%) participants were female. The distribution of SI is shown in Supplementary Figure 3. The mean SI index was 90.41 ± 24.04, and we categorized the population into four groups based on the quartiles of the SI (Table 1).

**Table 1:** Population characteristics by quartiles of SI.

**Variables**	**Quartiles of SI**			
	**Quartile 1**	**Quartile 2**	**Quartile 3**	**Quartile 4**
	**(≤74.03)**	**(74.04–87.27)**	**(87.31–103.69)**	**(≥103.70)**
Participants, *N*	1286	1286	1286	1287
Age, years	66.74 (4.87)	66.43 (4.82)	66.39 (4.54)	66.60 (4.92)
Male, *N* (%)	590 (45.88%)	620 (48.21%)	717 (55.75%)	737 (57.26%)
Hypertension duration, years				
≤5	800 (62.21%)	1024 (79.63%)	942 (73.25%)	1009 (78.46%)
>5 to ≤10	146 (11.35%)	77 (5.99%)	180 (14.00%)	179 (13.92%)
>10	340 (26.44%)	185 (14.39%)	164 (12.75%)	98 (7.62%)
Heart rate, bpm	79.68 (9.14)	80.23 (8.99)	79.68 (9.20)	80.12 (9.11)
SBP, mmHg	141.43 (17.77)	140.76 (16.63)	140.23 (17.06)	139.03 (17.38)
DBP, mmHg	84.61 (12.66)	86.24 (12.66)	87.31 (12.62)	89.36 (12.54)
BMI, kg/m^2^	24.34 (2.37)	24.44 (2.40)	24.30 (2.33)	24.59 (2.36)
Current smoker, *N* (%)	509 (39.58%)	443 (34.45%)	391 (30.40%)	229 (17.81%)
Current drinker, *N* (%)	482 (37.48%)	381 (29.63%)	322 (25.04%)	180 (14.00%)
Comorbidities, *N* (%)				
Coronary heart disease	225 (17.50%)	197 (15.32%)	209 (16.25%)	220 (17.09%)
Diabetes	326 (25.35%)	384 (29.86%)	363 (28.23%)	361 (28.05%)
Dyslipidemia	799 (62.13%)	807 (62.75%)	771 (59.95%)	783 (60.84%)
Atrial fibrillation	39 (3.03%)	43 (3.34%)	42 (3.27%)	31 (2.41%)
Charlson comorbidity index				
0	415 (32.27%)	577 (44.87%)	629 (48.91%)	673 (52.29%)
1	414 (32.19%)	368 (28.62%)	335 (26.05%)	344 (26.73%)
≥2	457 (35.54%)	341 (26.52%)	322 (25.04%)	270 (20.98%)
Laboratory parameters				
ALT, U/L	21.87 (14.00–31.62)	23.00 (14.36–34.02)	23.98 (15.00–34.30)	25.20 (16.60–34.50)
AST, U/L	21.00 (16.00–28.00)	21.45 (16.89–27.64)	21.26 (16.39–27.28)	21.01 (16.00–27.66)
GGT, U/L	24.22 (15.14–36.60)	25.30 (16.00–38.58)	28.03 (17.94–40.14)	29.00 (19.00–42.22)
Creatinine, mg/dL	0.66 (0.13)	0.77 (0.17)	0.82 (0.19)	0.89 (0.20)
UA, μmol/L	303.68 (79.50)	325.87 (81.20)	334.40 (85.55)	349.45 (85.95)
BUN, mmol/L	5.29 (1.37)	5.17 (1.41)	5.30 (1.42)	5.26 (1.38)
Cystatin C, mg/L	1.07 (0.22)	0.96 (0.21)	0.87 (0.20)	0.75 (0.19)
eGFR, ml/min/1.73 m^2^	94.00 (17.75)	93.28 (18.18)	93.56 (17.64)	95.70 (18.54)
TC, mmol/L	4.47 (3.79–5.10)	4.52 (3.77–5.20)	4.52 (3.85–5.15)	4.47 (3.85–5.03)
TG, mmol/L	1.50 (1.06–2.15)	1.49 (1.06–2.25)	1.64 (1.11–2.32)	1.67 (1.15–2.41)
HDL-C, mmol/L	1.08 (0.92–1.26)	1.04 (0.89–1.23)	1.06 (0.89–1.24)	1.02 (0.88–1.21)
LDL-C, mmol/L	2.73 (2.15–3.27)	2.80 (2.20–3.32)	2.79 (2.24–3.33)	2.79 (2.26–3.26)
HbA1c, %	6.08 (0.91)	6.12 (0.90)	6.07 (0.94)	6.10 (0.89)
FPG, mmol/L	5.13 (1.13)	5.09 (1.11)	4.98 (0.98)	5.06 (1.13)
Hcy, μmol/L	14.26 (5.52)	14.62 (5.90)	15.33 (5.74)	15.36 (6.31)
Hs-CRP, mg/L	1.96 (1.10–3.34)	2.00 (1.13–3.46)	2.10 (1.18–3.46)	1.98 (1.11–3.18)
Medications, *N* (%)				
ACEI/ARB	969 (75.35%)	919 (71.46%)	916 (71.23%)	883 (68.61%)
Beta-blocker	495 (38.49%)	490 (38.10%)	459 (35.69%)	458 (35.59%)
Calcium channel blockers	1039 (80.79%)	1046 (81.34%)	1015 (78.93%)	1017 (79.02%)
Diuretic	292 (22.71%)	284 (22.08%)	320 (24.88%)	299 (23.23%)
Insulin	131 (10.19%)	152 (11.82%)	125 (9.72%)	121 (9.40%)
Oral antidiabetic drugs	196 (15.24%)	273 (21.23%)	230 (17.88%)	235 (18.26%)
Statins	597 (46.42%)	553 (43.00%)	633 (49.22%)	598 (46.46%)
Aspirins	901 (70.06%)	907 (70.53%)	883 (68.66%)	906 (70.40%)

Variables were presented as mean (SD), median (IQR), or *N* (%). Abbreviations: SI: sarcopenia index; SBP: systolic blood pressure; DBP: diastolic blood pressure; BMI: body mass index; ALT: alanine aminotransferase; AST: aspartate aminotransferase; GGT: gamma-glutamyl transferase; TC: total cholesterol; TG: triglyceride; HDL-C: high-density lipoprotein cholesterol; LDL-C: low-density lipoprotein cholesterol; HbA1c: hemoglobin A1c; FPG: fasting plasma glucose; Hcy: homocysteine; UA: uric acid; Cr: blood creatinine; BUN: blood urea nitrogen; hs-CRP: high-sensitivity C-reactive protein; eGFR: estimated glomerular filtration rate; ACEI: angiotensin-converting enzyme inhibitor; IQR: interquartile range; SD: standard deviation.

### Association of SI with total stroke and its subtypes

**Figure 1:**
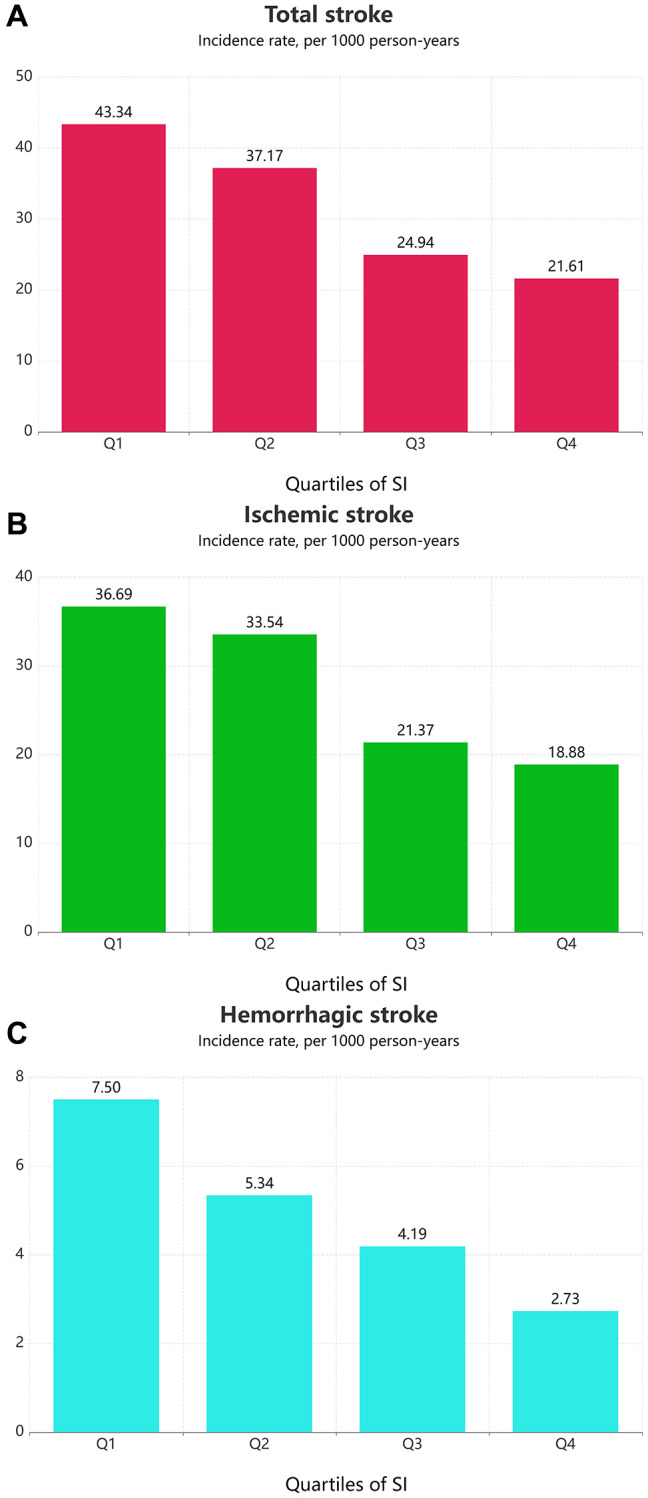
**Age-adjusted incidence of outcomes according to the SI quartiles.** (**A**) Total stroke; (**B**) Ischemic stroke; (**C**) Hemorrhagic stroke.

**Figure 2:**
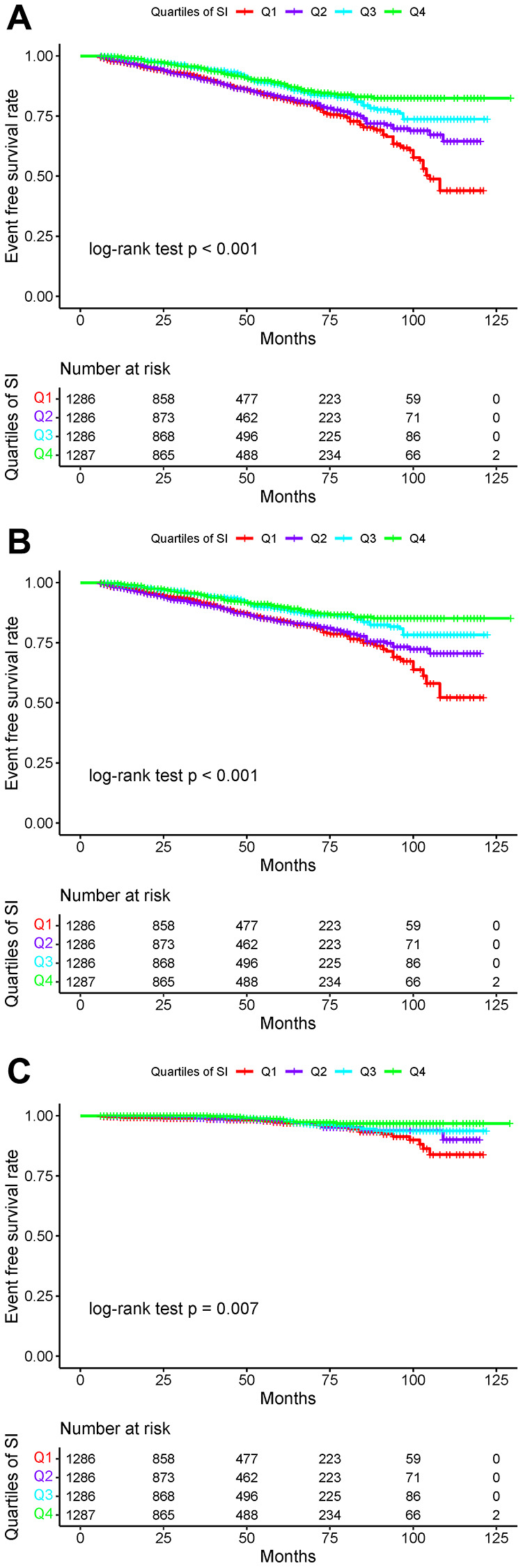
**Kaplan-Meier survival curves for total strokes and individual outcomes based on SI quartiles.** (**A**) Total stroke; (**B**) Ischemic stroke; (**C**) Hemorrhagic stroke.

**Figure 3:**
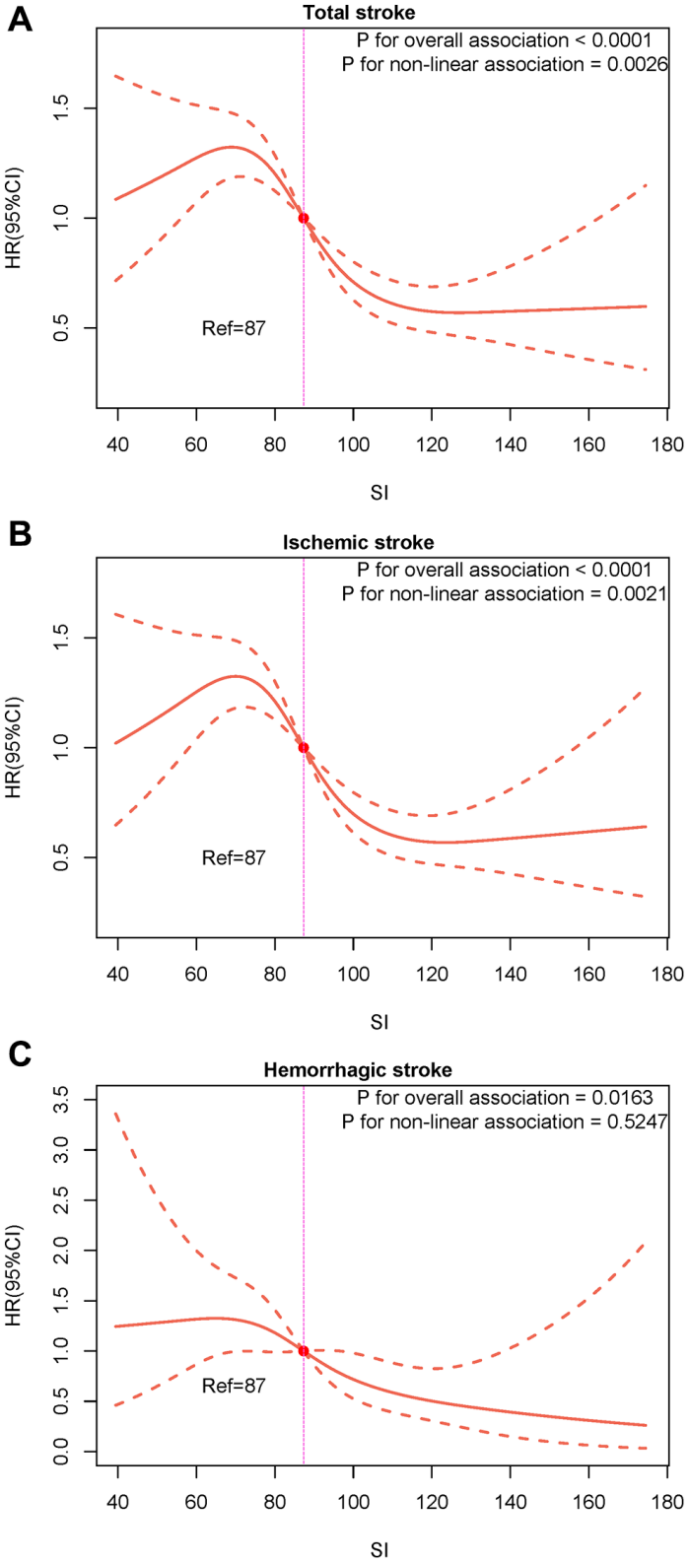
**Restricted cubic splines for the associations of SI with the risk of total stroke and its subtypes.** (**A**) Total stroke; (**B**) Ischemic stroke; (**C**) Hemorrhagic stroke.

Over a median follow-up of 38 months (IQR: 19–64 months), we identified 607 (11.80%) individuals with total stroke, of whom 507 (9.85%) had ischemic stroke and 93 (1.81%) had hemorrhagic stroke. The age-adjusted incidence of total strokes decreased substantially with the magnitude of SI (quartiles), reaching a maximum incidence of 43.34 per 1000 person-years in quartile 4 (Figure 1). Similarly, the risks of ischemic and hemorrhagic stroke decreased as SI quartiles increased. The Kaplan-Meier curve showed that participants in the quartile 1 group had a higher risk of total stroke, ischemic stroke, and hemorrhagic stroke than those in other groups (log-rank test, *P* < 0.001, Figure 2A; *P* < 0.001, Figure 2B; *P* = 0.007, Figure 2C). Overall, lower SI was significantly associated with higher hazards of stroke and its subtypes among elderly patients with hypertension (Figure 3). In the fully-adjusted model that measured the SI as a continuous variable, each 10-unit increment in the SI was associated with a 12% lower risk of total stroke (HR 0.88, 95% CI 0.85–0.92; Table 2). The cumulative hazard of total stroke also decreased with increasing SI, and this trend persisted even after adjusting for potential confounding factors in Model 3 (*P* for trend < 0.001). The HRs were 0.85 (95% CI, 0.69–1.05), 0.57 (95% CI, 0.45–0.72), and 0.46 (95% CI, 0.35–0.59) for the quartile 2, quartile 3, and quartile 4 groups versus the quartile 1 group of SI (Table 2). The results were similar when the association between SI and ischemic stroke and hemorrhagic stroke was examined (Table 2). In the restricted cubic spline analysis, we observed a significant dose-response relationship between SI and the risk of total stroke (*P* for non-linear association = 0.003) (Figure 3A). Similar results were observed in ischemic strokes (*P* for non-linear association = 0.002) (Figure 3B), but not in hemorrhagic strokes (*P* for non-linear association = 0.525) (Figure 3C).

**Table 2:** Association of SI with incident stroke.

**Outcome**	**HR (95% CI)**	
	**Model 1**	**Model 2**	**Model 3**
**Total stroke**			
Per 10-unit increment	0.89 (0.86, 0.92)	0.89 (0.86, 0.92)	0.88 (0.85, 0.92)
Quartiles			
Q1	Reference	Reference	Reference
Q2	0.86 (0.70, 1.05)	0.85 (0.69, 1.04)	0.85 (0.69, 1.05)
Q3	0.57 (0.45, 0.71)	0.58 (0.46, 0.73)	0.57 (0.45, 0.72)
Q4	0.49 (0.39, 0.63)	0.48 (0.37, 0.61)	0.46 (0.35, 0.59)
*P* for trend	<0.001	<0.001	<0.001
**Ischemic stroke**			
Per 10-unit increment	0.89 (0.86, 0.93)	0.89 (0.85, 0.93)	0.88 (0.85, 0.92)
Quartiles			
Q1	Reference	Reference	Reference
Q2	0.91 (0.73, 1.13)	0.90 (0.72, 1.12)	0.90 (0.73, 1.13)
Q3	0.58 (0.45, 0.74)	0.58 (0.46, 0.75)	0.58 (0.45, 0.74)
Q4	0.51 (0.40, 0.66)	0.49 (0.37, 0.63)	0.46 (0.35, 0.61)
*P* for trend	<0.001	<0.001	<0.001
**Hemorrhagic stroke**			
Per 10-unit increment	0.86 (0.79, 0.95)	0.86 (0.78, 0.95)	0.85 (0.77, 0.94)
Quartiles			
Q1	Reference	Reference	Reference
Q2	0.71 (0.42, 1.18)	0.71 (0.42, 1.20)	0.71 (0.42, 1.20)
Q3	0.55 (0.32, 0.95)	0.54 (0.31, 0.94)	0.52 (0.29, 0.93)
Q4	0.36 (0.19, 0.67)	0.35 (0.18, 0.68)	0.33 (0.17, 0.64)
*P* for trend	0.001	0.001	0.001

Model 1, adjusted for age and sex. Model 2, adjusted for variables in model 1 plus SBP, DBP, BMI, hypertension duration, heart rate, smoking status, drinking status, and comorbidities. Model 3, adjusted for variables in model 2 plus ALT, AST, GGT, UA, BUN, eGFR, TC, TG, HDL-C, LDL-C, HbA1c, FPG, Hcy, use of statins, use of aspirins, use of insulins, use of oral antidiabetic drugs, and antihypertensive drugs. Abbreviations: CI: confidence interval; HR: hazard ratio. Other abbreviations as presented in Table 1.

To investigate the robustness of our findings, we conducted multiple sensitivity analyses. In the sensitivity analyses, the associations of SI with the risk of stroke and its subtypes were not materially changed after excluding participants who developed strokes within the first year of follow-up (Supplementary Table 4), participants with CCI ≥ 2 (Supplementary Table 5), participants with atrial fibrillation (Supplementary Table 6), or participants with a competing risk model (Supplementary Table 7). Sensitivity analyses using E-values also revealed that strong unmeasured confounding is required for the observed association to be null (Supplementary Table 8 and Supplementary Figure 4).

### Stratified analyses

**Figure 4:**
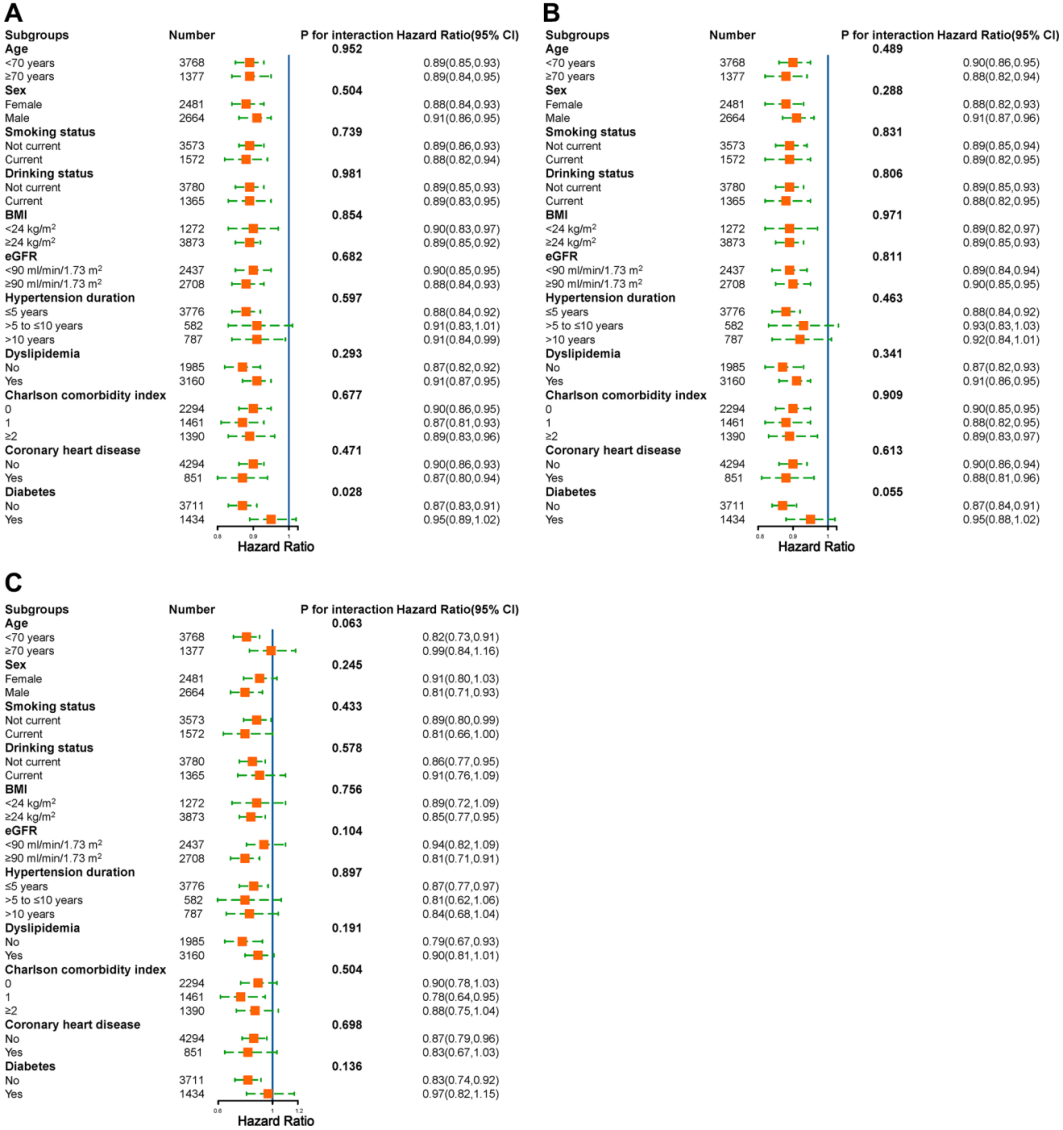
**Adjusted hazard ratios of stroke associated with per 10 unit increase in SI.** (**A**) Total stroke; (**B**) Ischemic stroke; (**C**) Hemorrhagic stroke.

We further performed exploratory subgroup analyses to assess the association between SI (per 10-unit increment) and the risk of stroke and its subtypes among elderly patients with hypertension (Figure 4). In the stratified analyses, age, sex, BMI, smoking status, drinking status, eGFR, hypertension duration, dyslipidemia, CCI, and coronary heart disease did not significantly modify the association between the SI and the risk of new-onset total strokes (all *P*-interactions > 0.05) (Figure 4A). Although the *P* value for the interaction of diabetes status in elderly hypertensive patients was less than 0.05, due to the similar directionality of the association, the result may not be clinically significant (Figure 4A). None of the variables significantly modified the association between SI and the risk of new-onset ischemic stroke and hemorrhagic stroke (all *P* for interactions > 0.05) (Figure 4B and 4C).

### Incremental predictive value of SI

We assessed whether SI would boost the traditional model’s prediction power even further (Supplementary Table 9). With the addition of SI, the conventional model’s C statistics greatly improved (Δ C-statistics = 0.02). With an IDI of 0.03 (95% CI, 0.02–0.04) and an NRI of 0.17 (95% CI, 0.10–0.23), the discriminating power and risk reclassification also seemed to be much superior. Both ischemic and hemorrhagic strokes had similar results.

## DISCUSSION

This study examined the relationship between SI and incident stroke in elderly hypertensive patients. After controlling for multiple confounders, the results showed that the risk of stroke was substantially associated with SI. Furthermore, in the restricted cubic spline analysis, we observed a significant dose-response relationship between SI and the risk of total strokes and ischemic strokes. These findings were consistent in both the sensitivity and subgroup analyses. Overall, the current investigation showed that low SI was associated with a higher risk of incident stroke, independent of other traditional risk variables. To our knowledge, this is the first large cohort study to demonstrate a link between SI and the risk of incident stroke among elderly hypertensive patients.

Currently, China has the world’s largest elderly population [32]. According to the results of the seventh national census in 2020, the population aged 60 and above accounted for 18.7% of China’s total population, amounting to 264 million people [33]. Population aging has become a significant trend in China’s social development, and the aging of China’s population has further intensified. In our aging society, sarcopenia, or reduced muscle mass, is a growing health concern [34]. Sarcopenia, which is a widespread and gradual reduction in skeletal muscle mass and function that impairs mobility and work capability in elderly people, is regarded as a geriatric syndrome [8]. Skeletal muscle degeneration, according to epidemiological research, starts after the age of 40 and gets worse with time. Skeletal muscle deteriorates in both number and quality at a rate of 8% each year [35]. The results of a recent meta-analysis showed that the prevalence of sarcopenia among the Chinese elderly aged 60 years and older was about 11.2% to 33.7% [36]. Stroke is the leading cause of disability and death among the elderly in China [37]. Some studies have linked skeletal muscle weakness to eating disorders, lack of exercise, insulin resistance, inflammation, and atherosclerosis and found that these are independent risk factors for cardiovascular disease in older adults [17, 38–41]. Sarcopenia imposes a significant burden on society by significantly increasing hospitalization and mortality rates in elderly patients [42, 43]. Screening for sarcopenia in elderly hypertensive patients at an early stage is therefore critical in clinical practice.

In this context, the quantification of skeletal muscle mass is particularly important. To assess skeletal muscle mass, several options have been proposed: magnetic resonance imaging, computed tomography, dual-energy X-ray absorptiometry, and bioelectrical impedance analysis [44]. However, these measurements are not universally applicable in clinical practice due to their high cost, possible radiation exposure, and the requirements for specialized technicians and sophisticated equipment [45]. Therefore, there is a need for more applicable and reliable alternative serum biomarkers for the assessment of sarcopenia in elderly patients with hypertension. Recently, the SI has been proposed as a simple alternative screening tool [23]. The results of numerous studies have suggested that the Cr/CysC ratio may serve as a marker for predicting muscle atrophy and dysfunction [44–48]. The Cr/CysC ratio has been positively correlated with muscle mass and strength in various populations [46, 49, 50]. In particular, the accuracy of the Cr/CysC ratio as a measure of muscle mass has been validated by computed tomography in the assessment of muscle mass in different populations, including the elderly and cancer patients [45, 51, 52]. Furthermore, evidence that the Cr/CysC ratio is superior to bioelectrical impedance analysis in the detection of muscle weakness further supports its accuracy [52].

In this study, we confirmed that SI was independently associated with an increased risk of stroke in elderly patients with hypertension. And we also observed a significant dose-response relationship between SI and the risk of total and ischemic strokes. Our findings are consistent with those of several previous studies. The difference is that previous studies have focused on patients with chronic renal insufficiency, older patients undergoing transcatheter aortic valve replacement, and patients with obstructive coronary artery disease [22, 53–55]. Further, they usually focus on looking at the risk of SI with cardiovascular death, all-cause death, and major adverse cardiovascular events. The underlying mechanism leading to this association may be multifactorial and has not yet been identified. Several potential explanations may account for this result. Skeletal muscle is a major site of glucose uptake, deposition, and actin secretion [56]. Therefore, the reduced glucose uptake caused by low skeletal muscle mass loss may contribute to enhanced insulin resistance [57, 58]. Insulin resistance can produce chronic hyperglycemia, which in turn triggers oxidative stress, causing an inflammatory response and cell damage [59]. Insulin resistance can also alter systemic lipid metabolism, leading to the development of dyslipidemia. This, combined with endothelial dysfunction, insulin resistance, and dyslipidemia, can all lead to atherosclerosis and eventually progress to ischemic stroke [60]. Increased muscle strength is associated with lower blood pressure and improved hemodynamics, suggesting a protective role for muscle in the development of atherosclerosis [61]. Another possible mechanism of poor prognosis is a decrease in skeletal muscle and endocrine function as secretory organs [62]. Myogenic factors are cytokines or other peptides produced, expressed, and released by skeletal muscle fibers that may help regulate beneficial cardiovascular effects [63]. Myocytes perform endocrine functions by secreting cardiovascular-beneficial myokines [64]. In patients with low muscle mass, decreased muscle cell numbers and decreased endocrine function may lead to adverse clinical outcomes [65]. These factors, together with pre-existing chronic comorbidities, explain the high risk of stroke. There are also studies showing that in older patients with skeletal myasthenia, long-term systemic chronic inflammation appears to be associated with the overall course of cardiovascular disease [66, 67]. Aging-related secretory phenotyping, one of the key factors in the chronic inflammation-induced instability of atherosclerotic plaques, is part of the pathogenesis of atherosclerosis, and is an independent risk factor for cardiovascular disease and cardiovascular mortality [66, 68, 69]. Research has also shown that inflammation activates the body’s catabolic pathway and promotes the hydrolysis of muscle proteins, leading to an imbalance between protein synthesis and catabolism, which further exacerbates the development of sarcopenia [70].

The present study has multiple strengths. Initially, this study is the first to use data from a cohort study to identify SI as a predictor of stroke in elderly patients with hypertension. Our findings should be considered in clinical practice and prospective clinical trials to prevent or treat pre-existing sarcopenia in older patients with hypertension. Second, we adjusted for as many confounders as possible in our study to improve the reliability of the results. Finally, this study used subgroup analysis and restricted cubic spline curve analysis, which helped enrich the interpretation of the relationship between SI and the risk of stroke. This study still has the following limitations: First, the observational, retrospective design limits the determination of causality. Second, we did not use methods such as dual energy X-ray absorptiometry or magnetic resonance imaging to assess actual residual muscle mass in these patients or analyze the association between SI and skeletal muscle mass, or sarcopenia. However, our goal was to focus more on the prognostic value of SI and obtain results that might be helpful in actual clinical practice. Third, we used measurements from only one point in time, so trends and changes in SI could not be determined. Fourth, this study was conducted only in hypertensive patients aged 60 years and older, and therefore our results may not be fully generalizable to younger people or other populations. Finally, although we examined measured covariates for potential confounding, residual confounding resulting from unmeasured factors such as frailty, physical activity, and dietary factors cannot be excluded.

In summary, this study found that elevated SI was negatively associated with the risk of stroke in elderly patients with hypertension. Uncovering the causality behind the relationship requires further prospective study.

## Supplementary Materials

Supplementary Materials and Methods

Supplementary Figures

Supplementary Tables
