# Supplementary material for: Association between the sarcopenia index and the risk of stroke in elderly patients with hypertension: a cohort study
Source: Aging (Albany NY). 2023 Mar 21;15(6):2005–32. doi: 10.18632/aging.204587 (PMC10085603; doi:10.18632/aging.204587)
Supplement: Supplementary Materials and Methods [file aging-15-204587-s001.pdf]

SUPPLEMENTARY MATERIALS AND METHODS

Details of the statistical analyses

To address the missing data in the covariates (Supplementary Table 2), we applied multivariate imputation using chained equations. We conducted 10 rounds of multiple imputations, then combined them into final estimates according to Rubin's rule (function "with/pool" in R package "mice"). The results of the analyses with imputation of missing variables were similar to those obtained from complete case analyses. Therefore, all analyses reported here were performed with multiple imputation of missing values. Continuous variables were expressed as mean ± standard deviation (SD) or medians with interquartile ranges depending on their distributions, and categorical variables were expressed as frequencies and percentages. Normality of datasets was tested using the KS or D'Agostino-Pearson omnibus normality test methods. To compare the characteristics among different SI groups, the chi-square test was performed for categorical variables, and one-way analysis of variance or the Kruskal-Wallis test was performed for continuous variables with normal and skewed distributions.

The age-adjusted incidence rates were determined by calculating age-specific incidence rates within 1-year age categories. The time to the first stroke event was examined using Kaplan-Meier survival curves and compared using the log-rank test. To assess for collinearity, we measured the variance inflation factor (VIF) in all models using a predetermined threshold of 5 as suggestive of multicollinearity (Supplementary Table 3). Variables with VIFs above 5 were removed. The proportional hazard assumptions were evaluated by visualization of Schoenfeld residuals, and no potential violation was observed (Supplementary Figure 2). Three multivariate Cox proportional hazard regressions were constructed to estimate the association of the SI with the risk of stroke by calculating the hazard ratio (HR) and 95% confidence interval (CI). In the first model, we adjusted for age and sex. The second model was adjusted for model 1 plus SBP, DBP, BMI, hypertension duration, heart rate, smoking status, drinking status, and comorbidities. The third model was adjusted for model 2 plus ALT, AST, GGT, UA, BUN, eGFR, TC, TG, HDL-C, LDL-C, HbA1c, FPG, Hcy, use of statins, use of aspirins, use of insulins, use of oral antidiabetic drugs, and antihypertensive drugs. In addition, we also assessed the associations of SI with stroke subtypes, including IS and HS. Trend tests were performed in the regression models after the median SI values of each quartile were entered into the model and treated as a continuous variable.

Additionally, restricted cubic splines were performed to examine the shape of the associations between SI and outcomes with five knots (at the 5th, 25th, 50th, 75th, and 95th percentiles). The reference point for SI was the median of the reference group, and the HR was adjusted for all confounding variables. The potential nonlinear relationships of SI with outcomes were explored.

In addition, possible modifications of the association between SI (per 10-unit increment) and outcomes were also assessed for the following variables: age (<70 or ≥70 years), sex (male or female), BMI (<24 or ≥24 kg/m^2), eGFR (<90 or ≥90 ml/min/1.73 m^2), current smokers (yes or no), current drinkers (yes or no), diabetes (yes or no), coronary heart disease (yes or no), CCI (0 or 1 or ≥2), hypertension duration (≤5 or 5–10 or >10 years), and hyperlipidemia (yes or no). Heterogeneity across subgroups was assessed by Cox proportional hazards models, and interactions between subgroups and SI were examined by likelihood ratio testing.

Sensitivity analyses were undertaken to evaluate the robustness of the results. First, to explore the potential impact of reverse causality, we repeated the primary analysis using a 1-year lag period, excluding participants who developed strokes within the first year of follow-up. Second, sensitivity analyses were also conducted to examine whether competing risks of non-stroke events were present. Third, sensitivity analyses were performed in subgroups from which all individuals with CCI ≥2 were excluded. Fourth, participants with atrial fibrillation were excluded. Lastly, potential unmeasured confounding was examined by calculating E-values.

Additionally, we used C-statistics, a net reclassification index (NRI), and an integrated discrimination improvement (IDI) to evaluate the incremental predictive value of the SI beyond the conventional model. The confidence intervals for the C-statistic, NRI, and IDI were computed by bootstrapping with 1000 resamples.

All analyses were completed in R version 4.1.1 (R Foundation for Statistical Computing), and all *P* values were two-sided.
