# Supplementary material for: Association between the sarcopenia index and the risk of stroke in elderly patients with hypertension: a cohort study
Source: Aging (Albany NY). 2023 Mar 21;15(6):2005–32. doi: 10.18632/aging.204587 (PMC10085603; doi:10.18632/aging.204587)
Supplement: Supplementary Figures [file aging-15-204587-s002.pdf]

SUPPLEMENTARY FIGURES

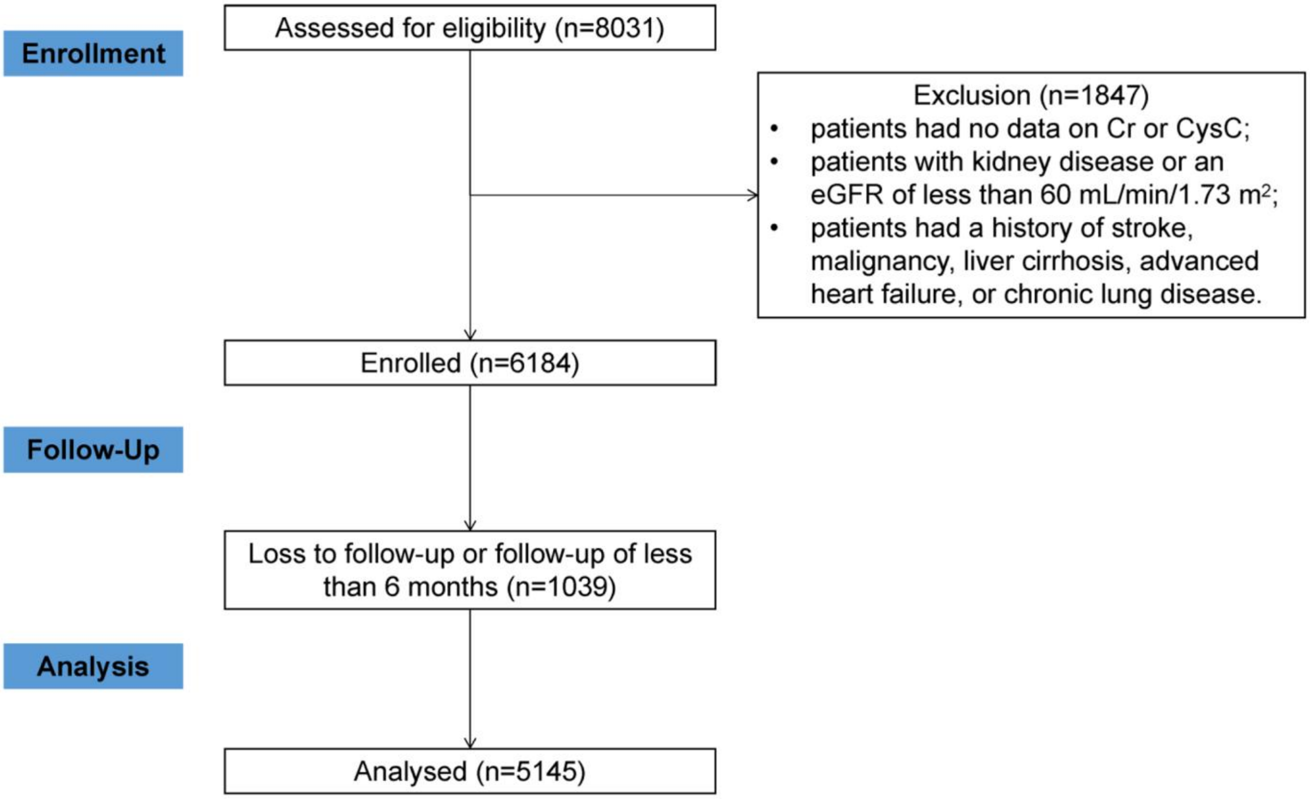

Supplementary Figure 1. Participant flow diagram.

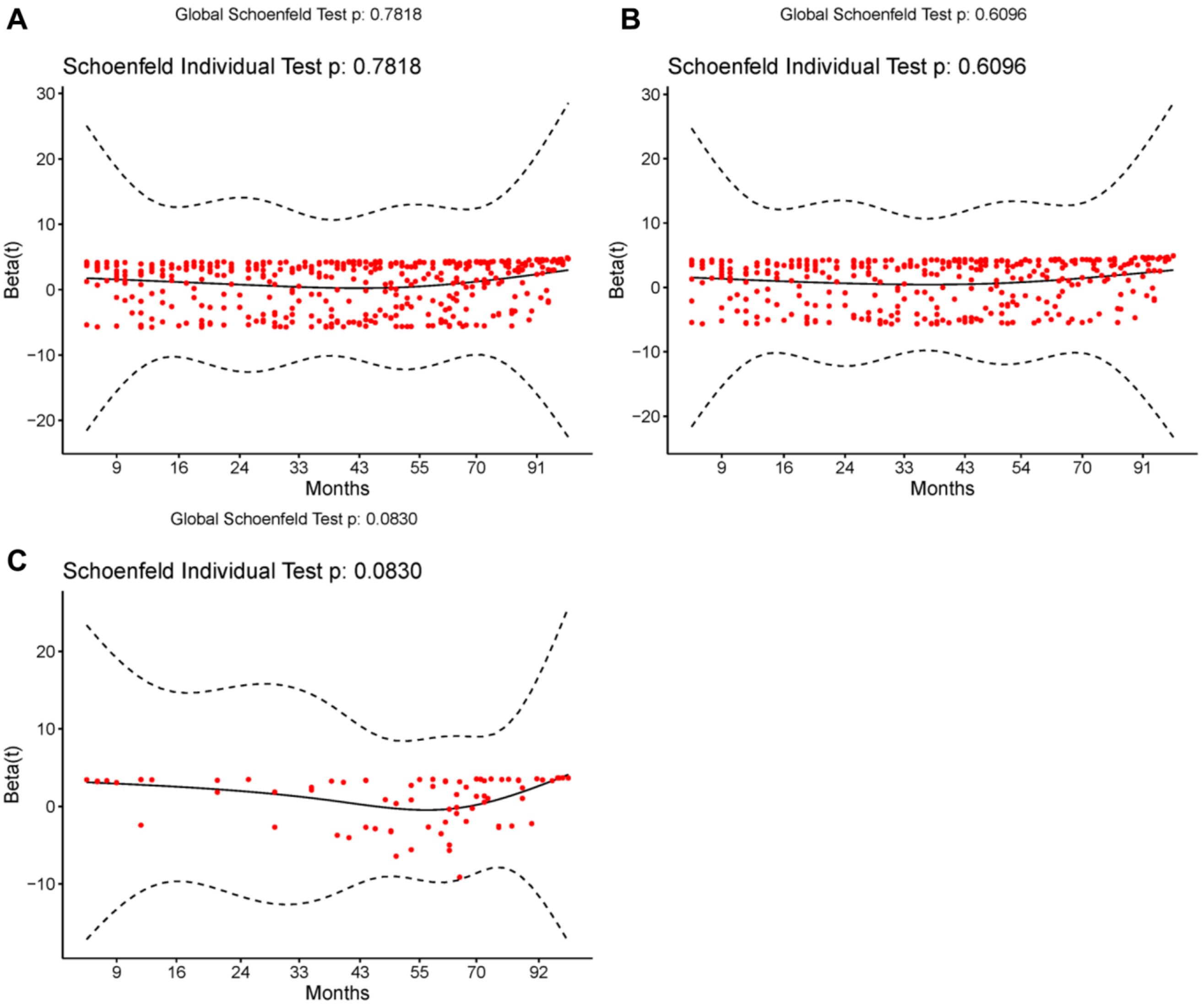

Supplementary Figure 2. Plot of Schoenfeld residuals against time in the Cox regression model. (**A**) Total stroke; (**B**) Ischemic stroke; (**C**) Hemorrhagic stroke.

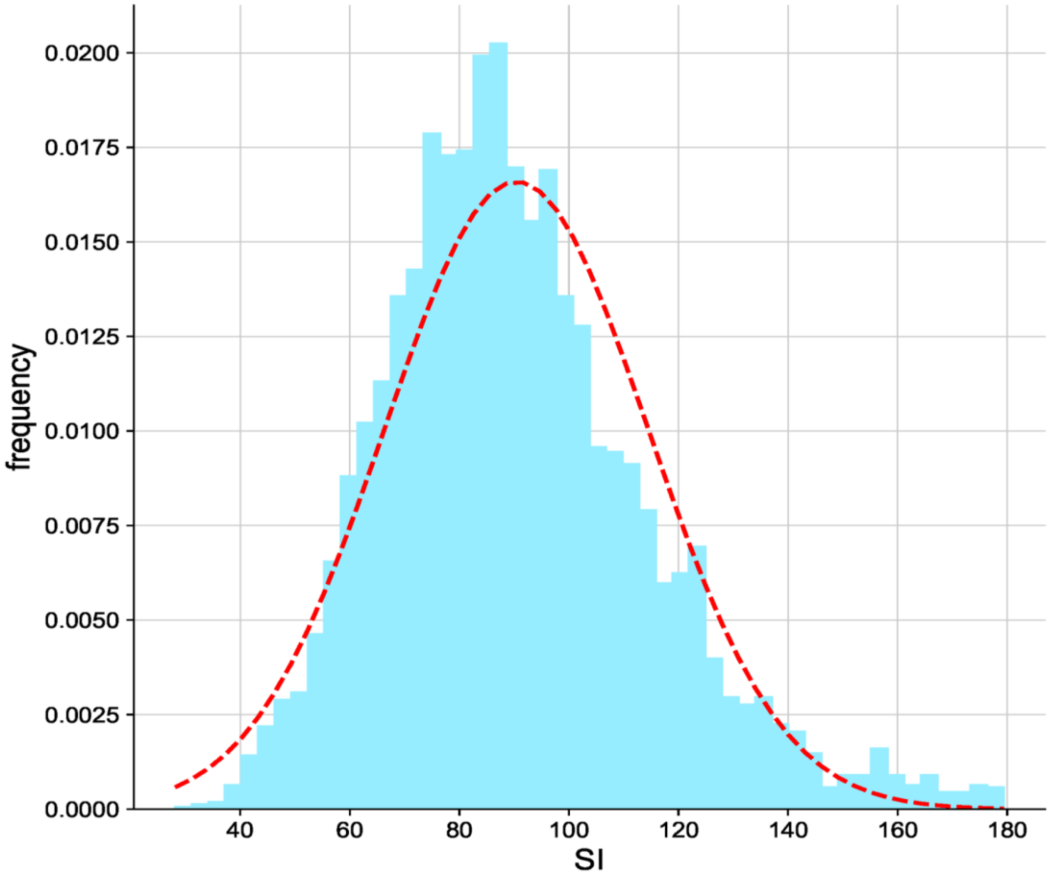

Supplementary Figure 3. Distribution of participants according to the SI.

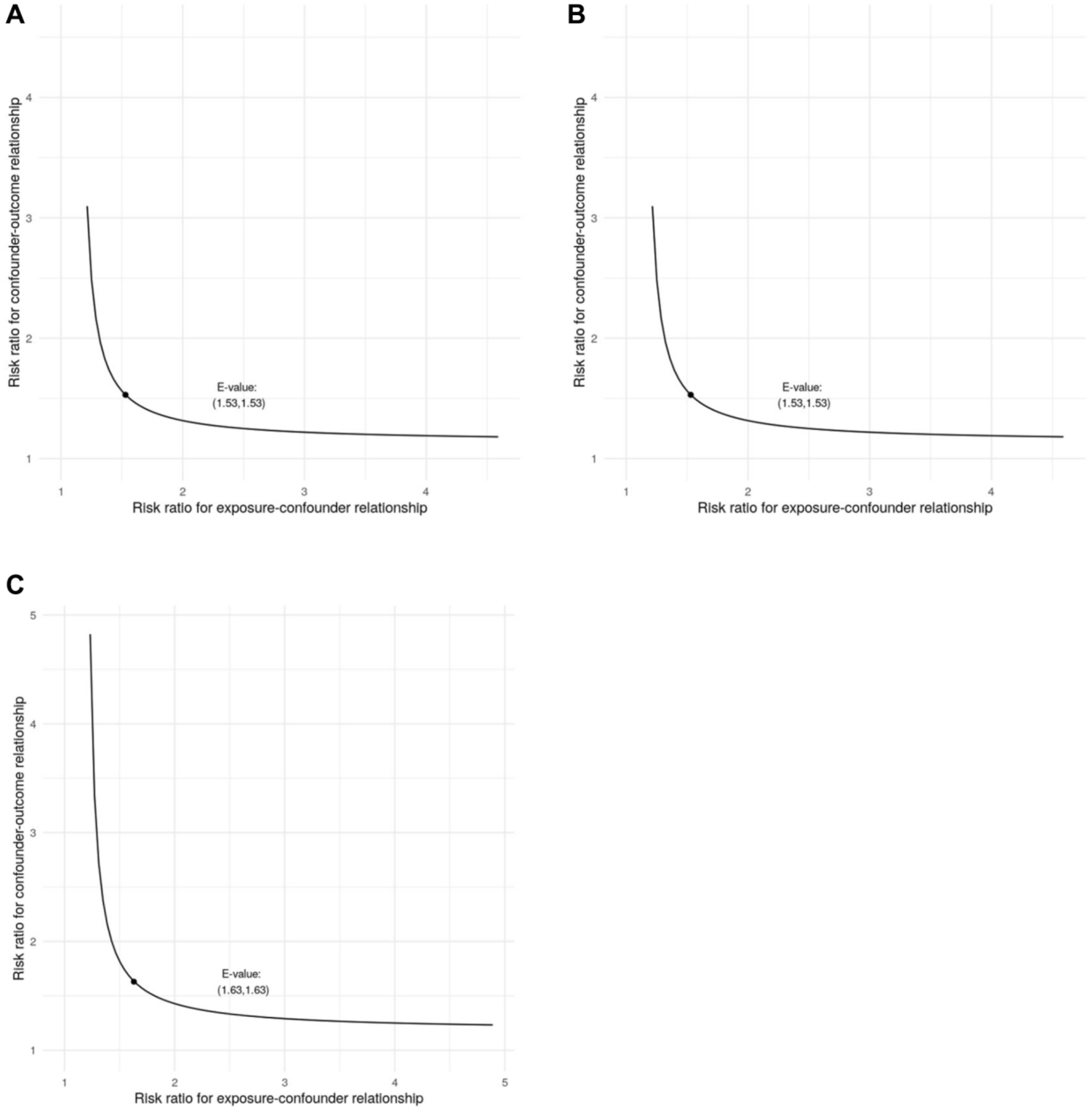

Supplementary Figure 4. E-values for the observed associations between SI and clinical outcomes. (**A**) Total stroke; (**B**) Ischemic stroke; (**C**) Hemorrhagic stroke.
