# Supplementary material for: Association between the sarcopenia index and the risk of stroke in elderly patients with hypertension: a cohort study
Source: Aging (Albany NY). 2023 Mar 21;15(6):2005–32. doi: 10.18632/aging.204587 (PMC10085603; doi:10.18632/aging.204587)
Supplement: Supplementary Tables [file aging-15-204587-s003.pdf]

SUPPLEMENTARY TABLES

Supplementary Table 1. List of medications included in the study.

Drug class	Drug name
Aspirin	Aspirin
Beta-blocker	Atenolol, bisoprolol, carvedilol, metoprolol, propranolol
Angiotensin-converting enzyme inhibitors or angiotensin receptor blockers	Azilsartan, candestartan, captopril, enalapril, fosinopril, irbesartan, losartan, olmesartan, ramipril telmisartan, valsartan
Calcium channel blockers	Amlodipine, diltiazem, felodipine, lercanidipine, nifedipine, verapamil
Diuretics	Acetazolamide, amiloride, benzyl hydrochlorothiazide, bumetanide, furosemide, hydrochlorothiazide, indapamide, spironolactone
Statin	Atorvastatin, fluvastatin, pitavastatin, rosuvastatin, simvastatin
Oral antidiabetic agents	Metformin, glipizide, gliclazide, glimepiride, glyburide, alogliptin, linagliptin, sitagliptin, vidagliptin, saxagliptin, acarbose, nateglinide, meglitinide, repaglinde, pioglitzone, dulaglutide, exenatide, liraglutide
Insulin	Rapid, short, intermediate and long-acting insulins

Supplementary Table 2. Counts and proportions of missing data.

Variables	Missing (%)
Heart rate	99 (1.92%)
SBP	103 (2.00%)
DBP	103 (2.00%)
BMI	84 (1.63%)
ALT	169 (3.28%)
AST	167 (3.25%)
GGT	154 (3.00%)
UA	148 (2.88%)
BUN	169 (3.28%)
TC	142 (2.76%)
TG	186 (3.62%)
HDL-C	124 (2.41%)
LDL-C	126 (2.45%)
HbA1c	245 (4.76%)
FPG	218 (4.24%)
Hcy	198 (3.85%)
Hs-CRP	398 (7.74%)

Abbreviations as presented in Table 1.

Supplementary Table 3. Collinearity diagnostics steps.

	Step 1	Step 2
SI	9.9	1.3
Sex	1.1	1.1
Age	1.0	1.0
HR	1.0	1.0
DBP	1.7	1.7
SBP	1.7	1.7
BMI	1.1	1.1
ALT	2.4	2.4
AST	2.1	2.1
GGT	1.4	1.4
Cr	**9.0**	**NA**
UA	1.5	1.5
BUN	1.1	1.1
Cystatin C	**10.5**	**NA**
eGFR	1.0	1.0
TC	4.2	4.2
TG	2.0	2.0
HDL-C	1.7	1.7
LDL-C	4.8	4.8
HbA1c	1.4	1.4
FPG	1.1	1.1
Hcy	1.1	1.1
Hs-CRP	1.0	1.0
Hypertension duration	1.0	1.0
Dyslipidemia	1.0	1.0
Atrial fibrillation	1.0	1.0
Charlson comorbidity index	1.0	1.0
Coronary heart disease	1.0	1.0
Diabetes	1.4	1.4
Current smoker	1.7	1.7
Current drinker	1.6	1.6

VIF = $1/(1-R^2)$. VIF step-by-step screening method: Calculate the VIF of each variable. If the maximum VIF value ≥5, remove the variable with the maximum VIF value. Abbreviation: VIF: variance inflation factors. Other abbreviations as presented in Table 1.

Supplementary Table 4. Sensitivity analysis excluding outcome events within the first year of follow-up.

Outcome	HR (95% CI)		
	**Model 1**	**Model 2**	**Model 3**
**Total stroke**			
Per 10-unit increment	0.91 (0.87, 0.94)	0.90 (0.87, 0.94)	0.90 (0.86, 0.93)
Quartiles			
Q1	Reference	Reference	Reference
Q2	0.86 (0.69, 1.06)	0.86 (0.69, 1.07)	0.85 (0.68, 1.06)
Q3	0.62 (0.49, 0.79)	0.63 (0.50, 0.81)	0.62 (0.49, 0.80)
Q4	0.55 (0.43, 0.70)	0.53 (0.41, 0.68)	0.50 (0.39, 0.65)
P for trend	<0.001	<0.001	<0.001
**Ischemic stroke**			
Per 10-unit increment	0.91 (0.87, 0.94)	0.90 (0.86, 0.94)	0.89 (0.86, 0.93)
Quartiles			
Q1	Reference	Reference	Reference
Q2	0.89 (0.70, 1.12)	0.88 (0.70, 1.12)	0.88 (0.69, 1.11)
Q3	0.61 (0.47, 0.79)	0.63 (0.48, 0.82)	0.62 (0.47, 0.81)
Q4	0.55 (0.43, 0.72)	0.53 (0.40, 0.70)	0.50 (0.38, 0.67)
P for trend	<0.001	<0.001	<0.001
**Hemorrhagic stroke**			
Per 10-unit increment	0.90 (0.82, 0.99)	0.89 (0.81, 0.98)	0.88 (0.80, 0.97)
Quartiles			
Q1	Reference	Reference	Reference
Q2	0.79 (0.46, 1.36)	0.79 (0.46, 1.38)	0.78 (0.44, 1.36)
Q3	0.66 (0.37, 1.17)	0.63 (0.35, 1.13)	0.60 (0.33, 1.08)
Q4	0.43 (0.22, 0.83)	0.42 (0.21, 0.82)	0.38 (0.19, 0.76)
P for trend	0.009	0.008	0.004

Model 1, adjusted for age and sex. Model 2, adjusted for variables in model 1 plus SBP, DBP, BMI, hypertension duration, heart rate, smoking status, drinking status, and comorbidities. Model 3, adjusted for variables in model 2 plus ALT, AST, GGT, UA, BUN, eGFR, TC, TG, HDL-C, LDL-C, HbA1c, FPG, Hcy, use of statins, use of aspirins, use of insulins, use of oral antidiabetic drugs, and antihypertensive drugs. Abbreviations: CI: confidence interval; HR: hazard ratio. Other abbreviations as presented in Table 1.

Supplementary Table 5. Sensitivity analysis of excluding participants with CCI ≥ 2.

Outcome	HR (95% CI)		
	Model 1	Model 2	Model 3
**Total stroke**			
Per 10-unit increment	0.89 (0.86, 0.93)	0.89 (0.86, 0.93)	0.89 (0.85, 0.93)
Quartiles			
Q1	Reference	Reference	Reference
Q2	0.93 (0.73, 1.18)	0.92 (0.72, 1.17)	0.92 (0.72, 1.17)
Q3	0.64 (0.49, 0.83)	0.66 (0.51, 0.87)	0.65 (0.49, 0.85)
Q4	0.49 (0.37, 0.65)	0.49 (0.36, 0.65)	0.46 (0.34, 0.62)
P for trend	<0.001	<0.001	<0.001
**Ischemic stroke**			
Per 10-unit increment	0.89 (0.86, 0.93)	0.90 (0.86, 0.94)	0.89 (0.85, 0.93)
Quartiles			
Q1	Reference	Reference	Reference
Q2	0.97 (0.75, 1.24)	0.96 (0.74, 1.23)	0.96 (0.74, 1.24)
Q3	0.61 (0.46, 0.81)	0.63 (0.47, 0.85)	0.62 (0.46, 0.83)
Q4	0.52 (0.39, 0.69)	0.51 (0.38, 0.69)	0.48 (0.35, 0.66)
P for trend	<0.001	<0.001	<0.001
**Hemorrhagic stroke**			
Per 10-unit increment	0.86 (0.77, 0.96)	0.86 (0.77, 0.97)	0.86 (0.76, 0.97)
Quartiles			
Q1	Reference	Reference	Reference
Q2	0.95 (0.51, 1.78)	0.93 (0.49, 1.76)	0.93 (0.48, 1.77)
Q3	0.80 (0.41, 1.54)	0.82 (0.42, 1.62)	0.79 (0.40, 1.58)
Q4	0.27 (0.11, 0.67)	0.27 (0.11, 0.70)	0.25 (0.10, 0.66)
P for trend	0.005	0.010	0.007

Model 1, adjusted for age and sex. Model 2, adjusted for variables in model 1 plus SBP, DBP, BMI, hypertension duration, heart rate, smoking status, and drinking status. Model 3, adjusted for variables in model 2 plus ALT, AST, GGT, UA, BUN, eGFR, TC, TG, HDL-C, LDL-C, HbA1c, FPG, Hcy, use of statins, use of aspirins, use of insulins, use of oral antidiabetic drugs, and antihypertensive drugs. Abbreviations: CI: confidence interval; HR: hazard ratio. Other abbreviations as presented in Table 1.

Supplementary Table 6. Sensitivity analysis of excluding individuals with prevalent atrial fibrillation at baseline.

Outcome	HR (95% CI)		
	**Model 1**	**Model 2**	**Model 3**
**Total stroke**			
Per 10-unit increment	0.90 (0.86, 0.93)	0.89 (0.86, 0.93)	0.89 (0.85, 0.92)
Quartiles			
Q1	Reference	Reference	Reference
Q2	0.88 (0.72, 1.09)	0.88 (0.72, 1.09)	0.88 (0.71, 1.08)
Q3	0.59 (0.47, 0.75)	0.59 (0.47, 0.75)	0.59 (0.46, 0.75)
Q4	0.51 (0.40, 0.65)	0.49 (0.38, 0.63)	0.47 (0.36, 0.61)
P for trend	<0.001	<0.001	<0.001
**Ischemic stroke**			
Per 10-unit increment	0.90 (0.87, 0.93)	0.89 (0.86, 0.93)	0.89 (0.85, 0.93)
Quartiles			
Q1	Reference	Reference	Reference
Q2	0.95 (0.76, 1.18)	0.94 (0.75, 1.17)	0.94 (0.75, 1.17)
Q3	0.60 (0.47, 0.77)	0.60 (0.47, 0.78)	0.60 (0.46, 0.77)
Q4	0.53 (0.41, 0.69)	0.50 (0.38, 0.65)	0.48 (0.36, 0.63)
P for trend	<0.001	<0.001	<0.001
**Hemorrhagic stroke**			
Per 10-unit increment	0.87 (0.79, 0.95)	0.86 (0.78, 0.95)	0.86 (0.78, 0.95)
Quartiles			
Q1	Reference	Reference	Reference
Q2	0.73 (0.44, 1.23)	0.74 (0.44, 1.25)	0.73 (0.43, 1.24)
Q3	0.57 (0.33, 0.99)	0.55 (0.31, 0.98)	0.54 (0.30, 0.97)
Q4	0.36 (0.19, 0.68)	0.36 (0.18, 0.69)	0.34 (0.17, 0.66)
P for trend	0.001	0.001	0.001

Model 1, adjusted for age and sex. Model 2, adjusted for variables in model 1 plus SBP, DBP, BMI, hypertension duration, heart rate, smoking status, drinking status, and comorbidities. Model 3, adjusted for variables in model 2 plus ALT, AST, GGT, UA, BUN, eGFR, TC, TG, HDL-C, LDL-C, HbA1c, FPG, Hcy, use of statins, use of aspirins, use of insulins, use of oral antidiabetic drugs, and antihypertensive drugs. Abbreviations: CI: confidence interval; HR: hazard ratio. Other abbreviations as presented in Table 1.

Supplementary Table 7. Sensitivity analysis was conducted using the Fine-Gray competing risk model considering non-stroke deaths as competing risk events.

Outcome	SHR (95% CI)		
	**Model 1**	**Model 2**	**Model 3**
**Total stroke**			
Per 10-unit increment	0.91 (0.87, 0.94)	0.90 (0.87, 0.94)	0.89 (0.86, 0.93)
Quartiles			
Q1	Reference	Reference	Reference
Q2	0.92 (0.75, 1.13)	0.92 (0.75, 1.13)	0.92 (0.75, 1.13)
Q3	0.67 (0.53, 0.84)	0.68 (0.54, 0.86)	0.66 (0.52, 0.84)
Q4	0.54 (0.42, 0.69)	0.51 (0.40, 0.66)	0.49 (0.38, 0.64)
P for trend	<0.001	<0.001	<0.001
**Ischemic stroke**			
Per 10-unit increment	0.91 (0.87, 0.94)	0.90 (0.86, 0.94)	0.89 (0.86, 0.93)
Quartiles			
Q1	Reference	Reference	Reference
Q2	0.98 (0.78, 1.22)	0.97 (0.77, 1.20)	0.97 (0.77, 1.21)
Q3	0.67 (0.52, 0.86)	0.68 (0.53, 0.88)	0.66 (0.51, 0.86)
Q4	0.55 (0.43, 0.71)	0.52 (0.40, 0.68)	0.49 (0.37, 0.65)
P for trend	<0.001	<0.001	<0.001
**Hemorrhagic stroke**			
Per 10-unit increment	0.89 (0.81, 0.97)	0.89 (0.81, 0.98)	0.88 (0.80, 0.97)
Quartiles			
Q1	Reference	Reference	Reference
Q2	0.78 (0.46, 1.33)	0.81 (0.47, 1.38)	0.80 (0.47, 1.38)
Q3	0.70 (0.40, 1.22)	0.70 (0.39, 1.25)	0.66 (0.37, 1.19)
Q4	0.41 (0.21, 0.78)	0.42 (0.21, 0.81)	0.38 (0.19, 0.77)
P for trend	0.007	0.010	0.006

Model 1, adjusted for age and sex. Model 2, adjusted for variables in model 1 plus SBP, DBP, BMI, hypertension duration, heart rate, smoking status, drinking status, and comorbidities. Model 3, adjusted for variables in model 2 plus ALT, AST, GGT, UA, BUN, eGFR, TC, TG, HDL-C, LDL-C, HbA1c, FPG, Hcy, use of statins, use of aspirins, use of insulins, use of oral antidiabetic drugs, and antihypertensive drugs. Abbreviations: CI: confidence interval; SHR: subdistribution hazard ratio. Other abbreviations as presented in Table 1.

Supplementary Table 8. E-values for the observed associations between SI and clinical outcomes.

	Total stroke	Ischemic stroke	Hemorrhagic stroke
Observed association* (Per 10-unit increment)	0.88 (0.85, 0.92)	0.88 (0.85, 0.92)	0.85 (0.77, 0.94)
E-value for point estimate	**1.53**	**1.53**	**1.63**
E-value for confidence interval	1.39	1.39	1.32

*The observed associations are the fully adjusted hazard ratios (95% confidence intervals) shown in Table 2 and are presented here for reference.

Supplementary Table 9. Incremental predictive value of the SI.

Models	Δ C-statistics (95% CI)	NRI (95% CI)	IDI (95% CI)
**Total stroke**			
Conventional model	Reference	Reference	Reference
Conventional model + SI	0.02 (0.01–0.03)	0.17 (0.10–0.23)	0.03 (0.02–0.04)
**Ischemic stroke**			
Conventional model	Reference	Reference	Reference
Conventional model + SI	0.02 (0.01–0.04)	0.19 (0.13–0.25)	0.02 (0.01–0.04)
**Hemorrhagic stroke**			
Conventional model	Reference	Reference	Reference
Conventional model + SI	0.01 (0.01–0.02)	0.14 (0.02–0.27)	0.01 (0.01–0.03)

The conventional model was adjusted for age, sex, SBP, DBP, BMI, hypertension duration, heart rate, smoking status, drinking status, comorbidities, ALT, AST, GGT, UA, BUN, eGFR, TC, TG, HDL-C, LDL-C, HbA1c, FPG, Hcy, use of statins, use of aspirins, use of insulins, use of oral antidiabetic drugs, and antihypertensive drugs. Abbreviations: CI: confidence interval; NRI: net reclassification index; IDI: integrated discrimination improvement. Other abbreviations as presented in Table 1.
